# Rising sea levels and the increase of shoreline wave energy at American Samoa

**DOI:** 10.1038/s41598-024-55636-y

**Published:** 2024-03-02

**Authors:** Austin T. Barnes, Janet M. Becker, Kelley A. Tagarino, William C. O’Reilly, Mika Siegelman, Philip R. Thompson, Mark A. Merrifield

**Affiliations:** 1 Scripps Institution of Oceanography, University of California San Diego, La Jolla, CA USA; 2 Sea Grant College Program, University of Hawai‘i at Mānoa, Honolulu, HI USA; 3 American Samoa Community College, Pago Pago, American Samoa USA; 4 Department of Oceanography, University of Hawai‘i at Mānoa, Honolulu, HI USA; 5 Cooperative Institute for Marine and Atmospheric Research, University of Hawai‘i at Mānoa, Honolulu, HI USA

**Keywords:** Physical oceanography, Physical oceanography, Climate-change impacts

## Abstract

American Samoa is experiencing rapid relative sea level rise due to increases in global sea level and significant post-2009 earthquake land subsidence, endangering homes and critical infrastructure. Wave and water-level observations collected over a fringing reef at Faga‘itua Bay, American Samoa, in 2017 reveal depth-limited shoreline sea-swell wave heights over the range of conditions sampled. Using field data to calibrate a one-dimensional, phase-resolving nonhydrostatic wave model (SWASH), we examine the influence of water level on wave heights over the reef for a range of current and future sea levels. Assuming a fixed reef bathymetry, model results predict rising sea levels will escalate nearshore extreme water levels that are dominated by an increase in nearshore sea-swell wave heights. Model results provide insight into how and at what reef depths rising sea levels reduce reef capacity to dissipate wave energy, compounding shoreline threats. This study aims to bring increased attention to the immediate threats to American Samoa’s way of life, and to demonstrate the utility of SWASH for extrapolating wave transformation to future sea level.

## Introduction

**Figure 1:**
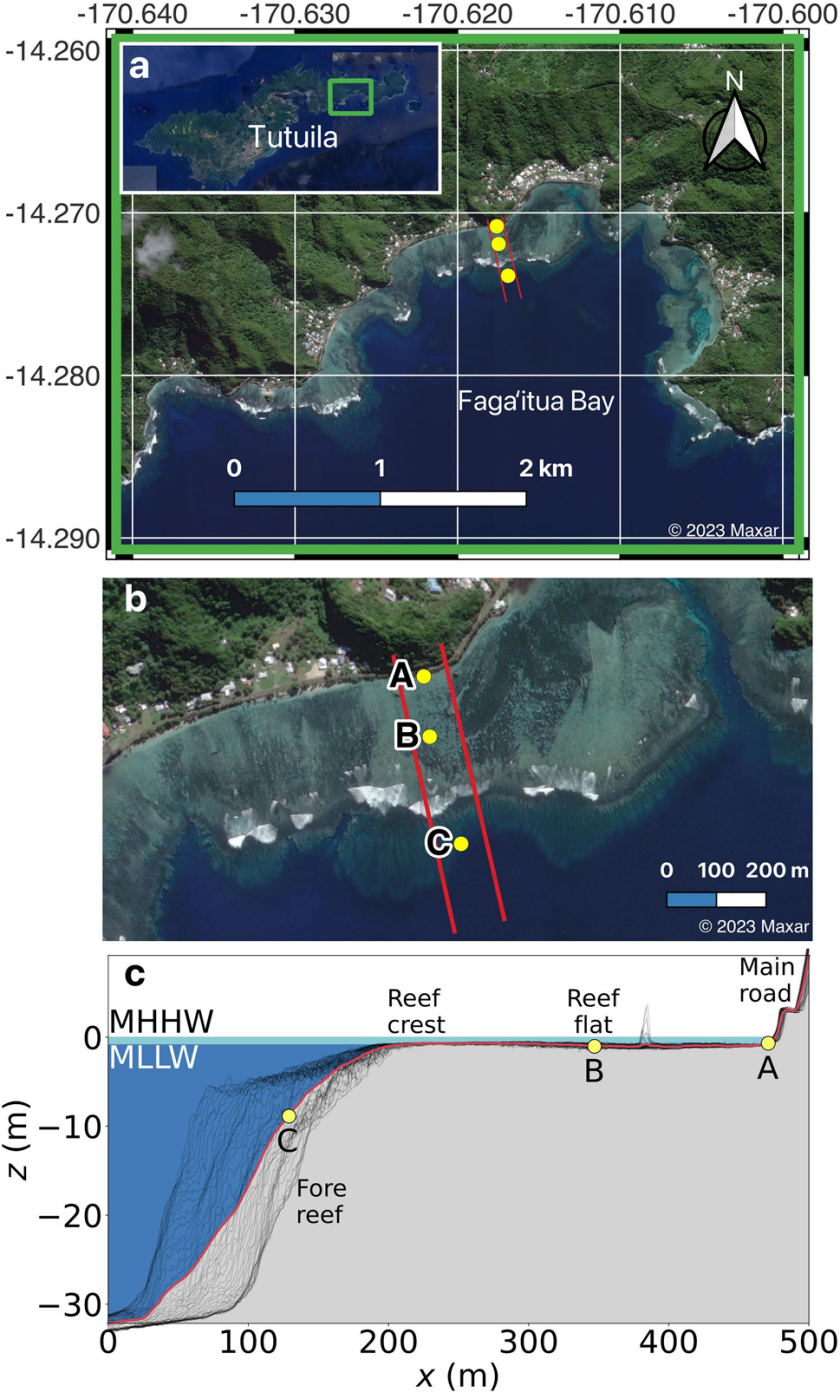
Faga‘itua Bay, Tutuila, American Samoa and cross-shore transect. (**a**) Satellite imagery of Tutuila Island (inset) and Faga‘itua Bay from (**b**) A closer view of the reef and its variable coral cover. Pressure sensors deployed in 2017 are marked in yellow (A = nearshore, B = mid-reef, C = reef face) and the red lines mark the 100 m-wide section of reef from which we derive an average reef transect. (**c**) Average reef bathymetry 1D transect (red) derived from transects spaced every 2 m in the alongshore direction (black). Mean higher high water (MHHW) and mean lower low water (MLLW) are indicated (tidal datum analysis period 01-Oct-2011 to 30-Sep-2019), as well as the sensor locations, fore reef, reef crest, reef flat, and the main coastal road.

American Samoa, currently an unincorporated United States territory in the South Pacific, is comprised of 5 main islands and 2 coral atolls, with Tutuila being the largest (142.3 km^2^), most populous (> 60,000) island (Fig. 1). Located near a semidiurnal tidal amphidrome, Tutuila experiences a small tidal range with 0.394 m mean high water (MHW) and 0.435 m mean higher high water (MHHW) above mean sea level (MSL)^1^. Similarly, semiannual (i.e., King Tides) and 4.4-year modulations of extreme high tides are weak^2^. Due to the low tidal variability and Tutuila's steep topography and narrow coastal plain, a majority of the population and infrastructure is located adjacent to the coastline, and is therefore highly vulnerable to storms, wave-driven flooding events, and sea level rise (SLR)^3^.

In September of 2009, a magnitude 8.1 Samoa-Tonga earthquake doublet caused a devastating tsunami and subsequent land subsidence. In 2013, the American Samoa Sea Grant extension agent noticed a distinct increase in sea level in National Oceanic and Atmospheric Administration’s (NOAA) Pago Pago tide gauge (1,770,000) record following the 2009 earthquakes^4^. Accelerated subsidence was confirmed for both Samoa and American Samoa^5^, and was subsequently federally recognized in 2019 following studies linking the subsidence to the 2009 earthquake doublet^6,7^. Based on NOAA’s Pago Pago tide gauge, satellite altimetry, and Global Navigation Satellite System land motion data, it was found that prior to the 2009 Samoa-Tonga earthquake doublet, land subsidence of Tutuila was occurring at an average rate of 1–2 mm/year. After the earthquakes, average rates of land subsidence increased to 16 mm/year during the period of 2009–2017^6^. While the rate of land subsidence has been slowing since 2009, it is expected to continue at well above pre-2009 rates for several decades due to viscoelastic relaxation, resulting in 30–40 cm of relative SLR this century, independent of global SLR^6^, equivalent to total SLR 4–5 times greater than the global average^8^. Updated relative SLR projections integrating future SLR for various socio-economic pathways provided by the United States Interagency Task Force (ITF) with land subsidence estimates were recently released for an American Samoa Sea Level Rise Viewer^9^. ITF estimates of SLR for American Samoa based on the intermediate emissions scenario would account for only 50% of total SLR by 2030, and by 2100 would underestimate total SLR by more than 0.2 m ^9^.

The main shoreline defense at Tutuila against energetic incident waves is a broad (up to 600 m), shallow (< 2 m) fringing reef that encompasses much of the island (Fig. 1). In general, coral reefs are highly efficient at dissipating incident sea-swell (SS) wave energy; e.g., Ferrario et al. (2014)^10^ estimate that reefs dissipate 97% of the incident energy ranging from typical to hurricane wave conditions. Field studies at islands with fringing reefs similar to Tutuila illustrate how waves break over the outer reef, with additional frictional dissipation over reef substrate at rates much higher than over sandy beaches^11^. In general, healthier reefs with greater reef canopy complexity have larger observed frictional dissipation rates^12^.

Energetic SS wave events carry the potential for severe impacts at island shorelines. Hoeke et al. (2013)^13^ describe a series of extreme coastal flooding events driven by swell from extratropical cyclones that impacted western Pacific islands in December 2008. In July 2022, the combination of King Tides and an energetic swell led to the worst coastal flooding since the 2009 Samoa-Tonga tsunami and was declared a major disaster by the U.S. Federal Emergency Management Agency (FEMA)^14^. Storlazzi et al. (2004)^7^ observed that greater water levels over a fringing coral reef off of Moloka‘i, Hawai‘i, allowed more of the deep-water SS energy to propagate over the reef flat, increasing the potential for shoreline erosion^15^. Becker et al. (2014)^16^ considered observations from three fringing reefs in the Pacific and found that water level over the reef flats controls the amount of shoreline SS wave energy. The dependence of SS shoreline wave heights on water level varies from site to site in part because the amount of dissipation depends on reef width and bottom roughness in addition to water depth over the reef flat.

While fringing reefs are effective at damping SS waves, longer period infragravity waves (25 to 200 s)^17^, far infragravity waves (200 to 1000 s)^18^, and wave setup^16^ can be energetic components of wave-driven shoreline water levels. Observations at Ipan reef, Guam, demonstrated that higher water levels increased the normal mode frequency of the fringing reef flat, and allowed for modal excitation of water levels in the far infragravity frequency band by wave-group forcing. The reef-flat modes have anti-nodes at the shoreline, resulting in increased low-frequency shoreline water level variation and therefore potential for flooding due to elevated water levels^18^. When the combination of reef geometry and water level does not lead to the excitation of reef modes, however, coral reefs have been demonstrated to reduce low-frequency motion on the reef flat and wave runup at the shoreline^19^.

Reef-fringed island shorelines differ from continental sandy beach shorelines in that runup amplitudes on sloping sand beaches increase with steepening beach slope, but not necessarily with water level. For example, the well-established statistical runup model of Stockdon et al. (2006)^20^ does not include a water level dependence, although beach slope may change with water level on non-uniform beach profiles. Climate change related projections of wave runup and coastal flooding on sandy beach shorelines thus depend on expected changes in the incident wave climate, e.g., due to changing storm patterns, or changes in shoreline morphology. The ability of island reefs to suppress wave energy is water-level dependent, and hence the influence of SLR may be twofold, causing both higher still water levels as well as higher wave energy. The inability of live reefs to accrete and keep pace with SLR is an additional potential concern. Absent other external stressors, relative SLR alone may potentially encourage reef growth, since reef accretion rates on shallow reef flats often are constrained by low tide and the lack of accommodation space^21^. Conversely, continued ocean warming and acidification will likely lead to coral bleaching and mortality, inhibiting reef accretion^22^. For the purposes of this study, we assume the reef bathymetry is fixed, and that relative SLR will increase the water depth over the fringing reef. Our projections are more closely tied to the water level over the fringing reef than to time, leaving room for the uncertainty in reef accretion and emissions pathway-dependent SLR.

Here we investigate how rising sea level over a fringing reef may affect nearshore sea-swell wave heights, lower frequency wave heights and breaking-wave setup, and highlight possible impacts of total water level changes on American Samoa. A goal of this work is to support calls for immediate mitigation and adaptation planning and lay the groundwork for forecasting future flooding events that result from the combination of elevated water level and swell waves. We use a one-dimensional, phase-resolving non-hydrostatic wave model to emulate wave transformation over a fringing reef study site for a range of present and future sea levels. The work extends a similar coastal flooding analysis conducted on reef-fringed shorelines in the Marshall Islands^23^; however, in this study the influence of increasing sea level on runup, and specifically SS wave energy, is explicitly modeled. As in Merrifield et al. (2014)^23^, we hindcast total water level to assess the influence of recent relative sea-level rise at American Samoa on total water levels. We examine the effects of land subsidence associated with the September 2009 Samoa-Tonga earthquake doublet. We focus on total water levels that are likely to reach the elevation of the existing coastal roadway that is the main transportation artery for Tutuila.

## Methods

### Field survey

Our study site is the fringing reef at Faga‘itua Bay, a southern-facing shoreline in eastern Tutuila and home to five villages (Fig. 1a). The Faga‘itua Bay morphology is representative of other embayed fringing reefs around the island. The reef varies in width from approximately 260 to 600 m, and in fore reef slope for an average slope of approximately 1/6 at our site. Spur and grooves on the fore reef extend up to the reef crest, which is still exposed at the lowest tides. The reef flat is shallow (< 2 m) and mainly reef rock. The shoreline around the bay is comprised of narrow (0–10 m) sandy beaches backed by rocky, vegetated slopes up to the main coastal road, which has a nominal elevation varying from 1.5 to 5 m (3.4 m at our transect location) above MSL. Wave breaking is typically confined to a narrow breaking zone at the outer edge of the reef flat.

From April to October 2017, three bottom-mounted pressure sensors (Sea-Bird SBE26plus) were deployed across the reef at Faga‘itua Bay. The sensors were located offshore of the steeply sloping shoreline in deployment-averaged water depths of 0.57 m near the shore (A), 1.00 m at mid reef (B), and 8.55 m on the steep fore reef (C) (Fig. 1b). Depths are referenced to MSL based on comparison with the National Oceanic and Atmospheric Administration (NOAA) Pago Pago tide gauge on Tutuila during the sampling period. To maximize battery life, measurements were collected at 1 Hz for 1.5 h bursts every 3 h. Intermediate water depth wave data from 2014 to present day is available from the Coastal Data Information Program’s (CDIP) buoy station 189 at Aunu‘u. At our study site, the sandy beach has been recently lost to rising relative sea level.

We convert pressure time series to surface elevation using first-order linear wave theory, from which we calculate wave spectra and bulk wave statistics. The 1.5-h bursts are broken into 30-min records and detided by removing the dominant M2 tidal component. Atmospheric pressure measurements collected every 6 min from NOAA's National Data Buoy Center (NDBC) station NSTP6—1770000 at Pago Pago are interpolated to match sensor samples and removed from total pressure measurements. For this study, we define the low frequency (LF) band, encompassing infragravity and far infragravity waves bands, from 0.001 to 0.04 Hz (periods of 25–1000 s) and the sea-swell (SS) band from 0.04 to 0.3 Hz (3.33–25 s). Records with water depth < 0.3 m are excluded from analysis. At Faga‘itua Bay, we have 2100 30-min records during which both the nearshore (A) and reef face (C) sensors were operational. The reef flat (B) sensor had issues with timing and stability during deployment, so we use the observations for verification of the bathymetric depth only.

We compute the nearshore breaking-wave setup following Becker et al. (2014)^16^ from the 30-min average water depths, ${\overline{h} }_{A}$ and ${\overline{h} }_{C}$. ${H}_{rms}^{A}$ and ${H}_{rms}^{C}$ are computed over each 30-min record as $2\sqrt{2}\sigma$, where $\sigma$ is the standard deviation from the surface elevation spectrum in the SS frequency band. We exclude setup calculations for weak wave conditions (${H}_{rms}^{C}-1.2{H}_{rms}^{A}$<0.4 m) when wave breaking is not expected to occur. For modeled setup, we use the default SWASH setup computation relative to the still water level (SWL) as defined by the initial water surface.

We compute the peak period by finding the peak frequency of the 1-dimensional wave spectrum, and the mean period following the National Data Buoy Center^24^ as

1 $${T}_{mean}={\left(\frac{{m}_{0}}{{m}_{2}}\right)}^\frac{1}{2}$$

2 $${m}_{0}=\sum S\left(f\right)d(f)$$

3 $${m}_{2}=\sum S\left(f\right){f}^{2}d(f)$$

where ${m}_{0}$ and ${m}_{2}$ are the zeroth- and second-order moments of the nondirectional wave spectra computed by summing the spectral density, $S\left(f\right)$, or $S\left(f\right){*f}^{2}$ over all spectral frequencies, $f$, respectively. The NDBC refers to this as the zero-crossing period in some references. With only pressure sensors we did not measure wave direction; however, based on visual observations we assume shore-normal incidence of waves and insignificant reflection of wave energy.

### Bathymetry

We specify a 1-m resolution cross-shore bathymetry transect at Faga‘itua using a preliminary digital elevation model (DEM) provided by NOAA. We verify that the reef bathymetry at Faga‘itua appears to closely match aerial photographs of alongshore variations including reef channels and smaller spur and groove features on the fore reef. Additionally, the relative depths of the 3 reef sensors match estimates of the mean water depths from observations to within 0.3 m. For the numerical modeling described in subsequent sections, we use a 500-m long, 1-dimensional grid from 32-m water depth to 10 m above MSL. We vary the cross-shore grid spacing from 2 m at the seaward boundary to 0.04 m at the shoreward boundary using linear interpolation. The input bathymetry has 3 grid points in the alongshore direction with 1-m spacing, and each cross-shore transect is identical. This single transect is derived from an average of parallel cross-shore transects spanning 100 m in the alongshore, spaced every 2 m (Fig. 1c). Several transects include a rock protrusion but its impact on the final average transect are negligible. We adjust the simulated still water level for each model run to match the reef face (sensor C) location water depth with the 30-min average water level for that observation period.

### 1-D Numerical modeling of nearshore waves using SWASH

Wave transformation across the Faga‘itua reef is simulated by the phase-resolving non-hydrostatic wave model Simulating WAves till SHore (SWASH) version 10.01. SWASH has been used to investigate waves over reefs with validation using laboratory experiments^25–27^ and field observations^28–31^. SWASH is a nonhydrostatic wave-flow model using the Reynolds-averaged Navier–Stokes Eqs. ^32^. We implement SWASH on a 1-dimensional transect but use the 2-dimensional, nonhydrostatic mode of SWASH to employ variable cross-shore grid spacing described above, following Fiedler et al. (2018)^33^. We use 10 vertical layers and the default breaking wave parameter^25^. Turbulent mixing was activated using the vertical $k-\epsilon$ model^34^ with default parameters. A background viscosity of $1*{10}^{-4}$ is assumed. Following Risandi et al. (2020)^28^, who studied a fringing reef in southwestern Australia with visually similar coral cover, we account for friction between the flow and rough bed using a constant friction factor of 0.05. SWASH is run with 15 min of spin-up time to allow breaking-wave setup to stabilize followed by 30 min of model time. We analyze the simulated sea surface elevation to compute wave spectra and significant wave heights in both the LF and SS frequency bands following the same methods used for the observations. The default SWASH method is used for computing wave setup.

At the offshore boundary we initiate simulations with 30-min average wave spectra computed from the reef face (C) sensor observations, back-refracted from observation depth (~ 8 m) to the model boundary depth (32 m) using linear wave theory. We neglect SS wave energy associated with reflections at the fore reef based on the findings of Péquignet et al. (2011)^35^ for a similar steeply sloping reef face. Time series of SS waves at the boundary are obtained from the back-refracted wave spectra assuming random wave phase. Of the 2100 observational records, a sample of 200 was chosen for numerical simulation and comparison. Of the 200 records chosen for the sample, 49 are the largest observed nearshore (A) water level records with ${\overline{h} }_{A}>0.9$ m to ensure that we compare model behavior at the highest observed water levels. The remaining 151 are randomly chosen to span the range of observed water levels and incident wave energy. Each of the 200 30-min incident wave spectra are simulated using SWASH with observed water levels for model validation.

The validated model is extended to higher water levels by maintaining the wave and tide conditions used in the historical hindcasts and increasing the still water level (SWL) (e.g., due to tides, non-tidal residual sea level, SLR, and vertical land motion) to 0.6–2.0 m above current MSL. The range of SLR is chosen to extend observed water levels to end of century projections for American Samoa made by Baizeau et al. (2023)^9^, based on a combination of sea level rise resulting from the ITF intermediate emissions scenario and vertical land motion estimates from Han et al. (2019)^6^. Baizeau et al. (2023)^9^ project 0.8249 m of relative SLR by 2070 and 1.4112 m by 2100, relative to 2005 MSL. We track the modeled runup line with a threshold depth of at least 0.01 m.

### Nearshore setup and sea-swell wave hindcasts

We perform a wave hindcast (January 1979 through May 2023) at Faga‘itua Bay using observed water levels at the Pago Pago tide gauge, a deep water wave hindcast, and a refraction model over local bathymetry to estimate reef face (sensor C) significant wave height. Empirical relationships then are used to estimate breaking-wave setup and nearshore SS wave height (sensor A).

We obtain a wave hindcast dating back to 1979 for the reef face location (sensor C) using an open-source WWIII-generated wave hindcast^36^ and a linear spectral refraction model^37^. The WWIII hindcast provides hourly directional spectra around American Samoa. We obtain spectra from the output model grid point located at −14.53°N, 189.53°E, just to the south of the CDIP Aunu‘u buoy, against which we validate the wave hindcast for periods of overlap. We use a non-stationary, linear spectral refraction wave model^37^ to generate a transfer matrix that is both a function of frequency and wave direction. The matrix is constrained with a cutoff of 130 degrees based on the geometry of Faga‘itua Bay to prevent errant excess energy making it to our reef face location. We bias-correct the model using our observations from the 2017 field deployment. The refracted hindcast overpredicts some of the local high-frequency seas observed, and misses some other observed events, but overall has reasonable skill in matching observations (${R}^{2}=0.63$, $RMSE=0.18$ m) from the 2017 field deployment.

We use observations from the 2017 field deployment to parameterize the nearshore breaking-wave setup as a function of the tide and the reef face significant wave height. As the observed setup at sensor A exhibits both a tidal and reef face wave height dependence, we follow Becker et al. (2014)^16^ to estimate a tidally dependent ($h^{\prime}$, MSL) factor,

4 $$p\left({h}^{\prime}\right)=0.10{h}^{{\prime}2}-0.12{h}^{\prime}+0.26$$

that, when multiplied by the reef face SS significant wave height, ${H}_{SS}^{C}$, provides an estimate of breaking-wave setup as

5 $${\widehat{\overline{\eta }}}_{A}=p\left({h}^{\prime}\right){H}_{SS}^{C}$$

A quadratic fit results in improved predictive skill (${R}^{2}=0.87$, $RMSE=0.02 m$) compared to a linear fit (${R}^{2}=0.45$, $RMSE=0.02 m$). This relationship is not meant to be extrapolated into higher sea levels beyond those observed (~ 0.7 m, MSL), but is useful for obtaining hindcast estimates of breaking-wave setup given observed water levels and hindcast wave heights.

To estimate historical nearshore breaking-wave setup using the regression fit (5), we use estimates of the reef face SS significant wave height, ${H}_{SS}^{C}$, computed using the wave hindcast. We then combine the observed sea level record from the Pago Pago tide gauge with our estimates of setup to obtain a total nearshore mean water level. We use model results discussed in the next section to estimate the nearshore SS significant wave heights from January 1979 through May 2023.

## Results

**Figure 2:**
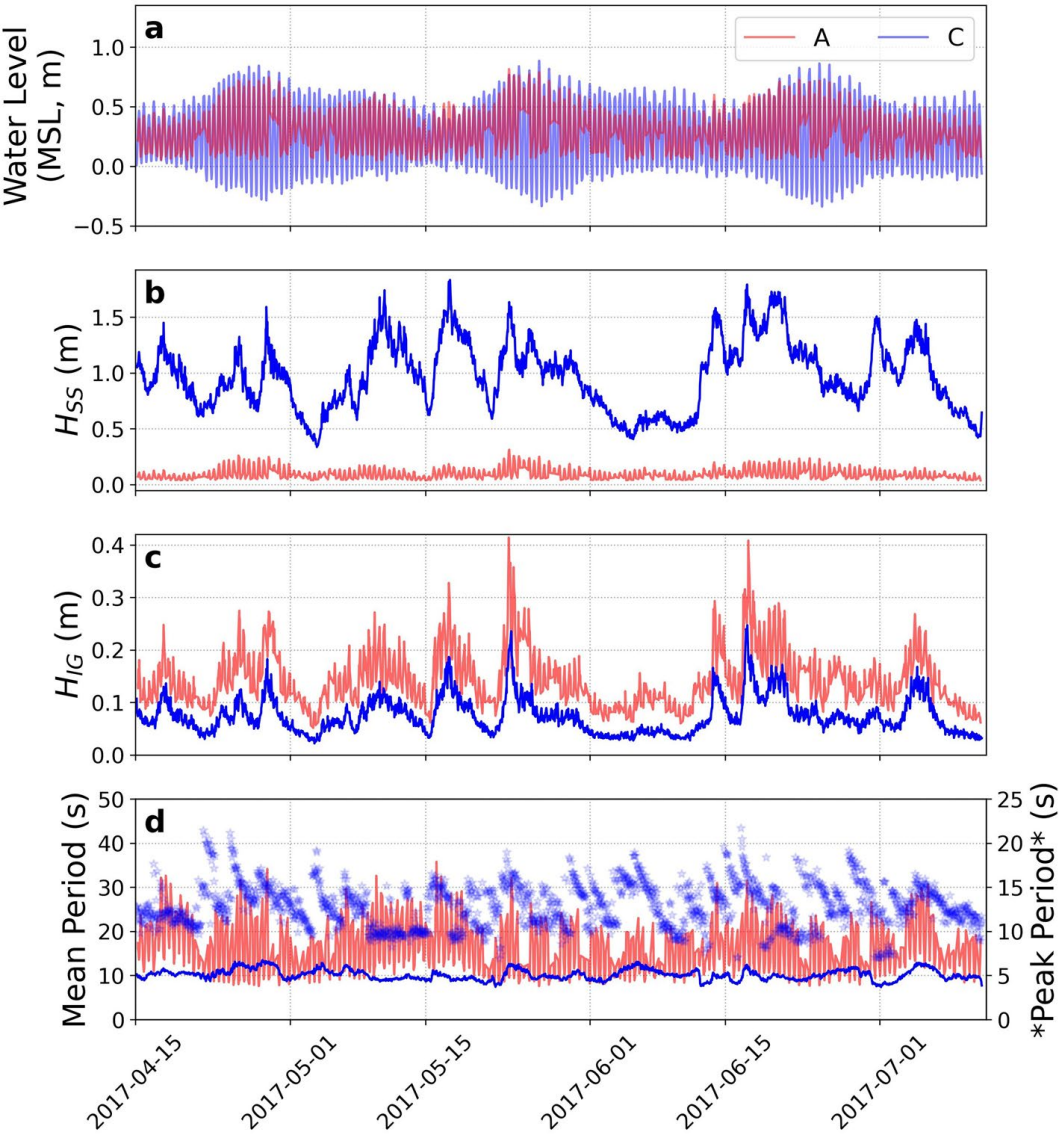
Observations from 2017 field deployment. Time series from sensor A (nearshore) and C (reef face) of: (**a**) 30-min average water level (MSL); (**b**) SS (0.04–0.3 Hz) significant wave height; (**c**) LF (0.001–0.04 Hz) significant wave height; (**d**) mean period (and peak period for sensor C only, shown in symbols).

The wave field at Faga‘itua reef exhibits strong SS energy dissipation and LF energy propagation across the reef flat (Fig. 2). Reef face (sensor C) SS significant wave heights (${H}_{SS}$) range between 0.3 and 1.8 m, while nearshore (sensor A) ${H}_{SS}$ never exceed 0.3 m (Fig. 2b). Nearshore LF significant wave heights often equal or exceed the SS counterpart, and nearshore LF energy exceeds the LF energy observed at the reef face (Fig. 2c,d).

**Figure 3:**
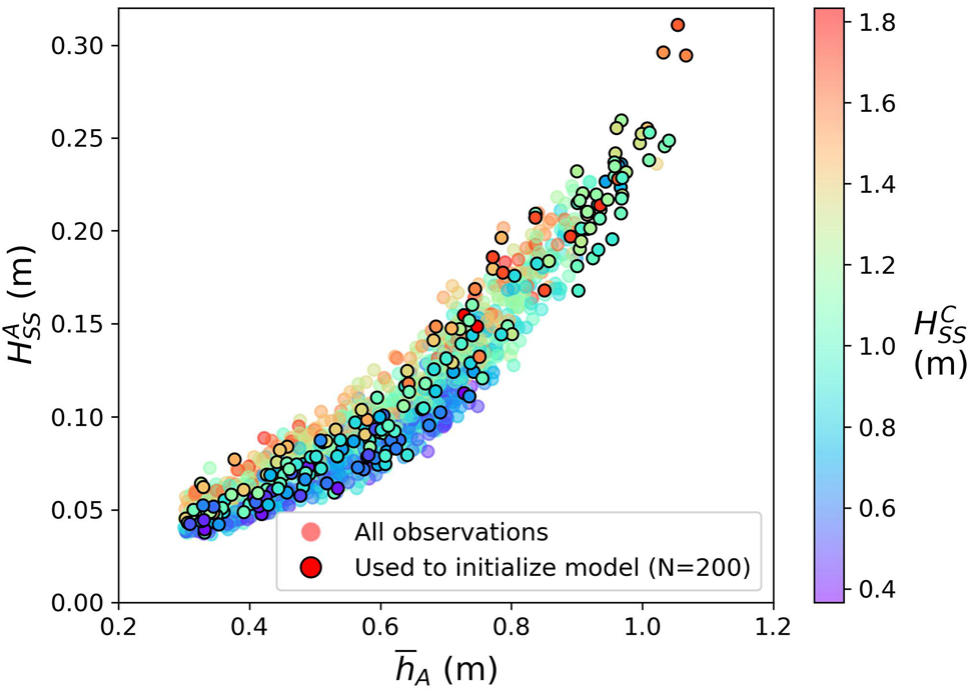
Nearshore (sensor A) sea-swell significant wave height as a function of mean water depth, colored by the observed significant wave height on the reef face (sensor C). All 30-min average observations with water depth > 0.3 m are shown; the 200 chosen for SWASH validation are marked with black borders. Nearshore sea-swell significant wave height is strongly depth-dependent, with weaker dependence on the reef face significant wave height.

The nearshore ${H}_{SS}$ scales with the mean water depth over the reef flat (Fig. 3), similar to the reefs studied for example in Becker et al. (2014)^16^. Nearshore ${H}_{SS}$ are depth limited for the considered range of offshore wave conditions at Faga‘itua. The depth limitation is set as waves break at the outer reef, i.e., waves break when their heights exceed a certain fraction of the water depth. Additional dissipation occurs as waves travel shoreward of the break zone due to bottom friction over the rough reef substrate and turbulent energy loss in the broken wave rollers, which both depend to some extent on water depth. The water level variations on the reef in turn are set by the tide, non-tidal residual sea level effects, and wave setup. For a given reef flat water level, the nearshore ${H}_{SS}$ exhibits a dependence on the offshore wave height; however, this effect is weak compared to the water-level dependence. As in Merrifield et al. (2014)^23^, observed nearshore ${H}_{SS}$ may be estimated using a quadratic fit to mean water level, but to what extent may this be extrapolated to higher, unobserved water levels?

**Figure 4:**
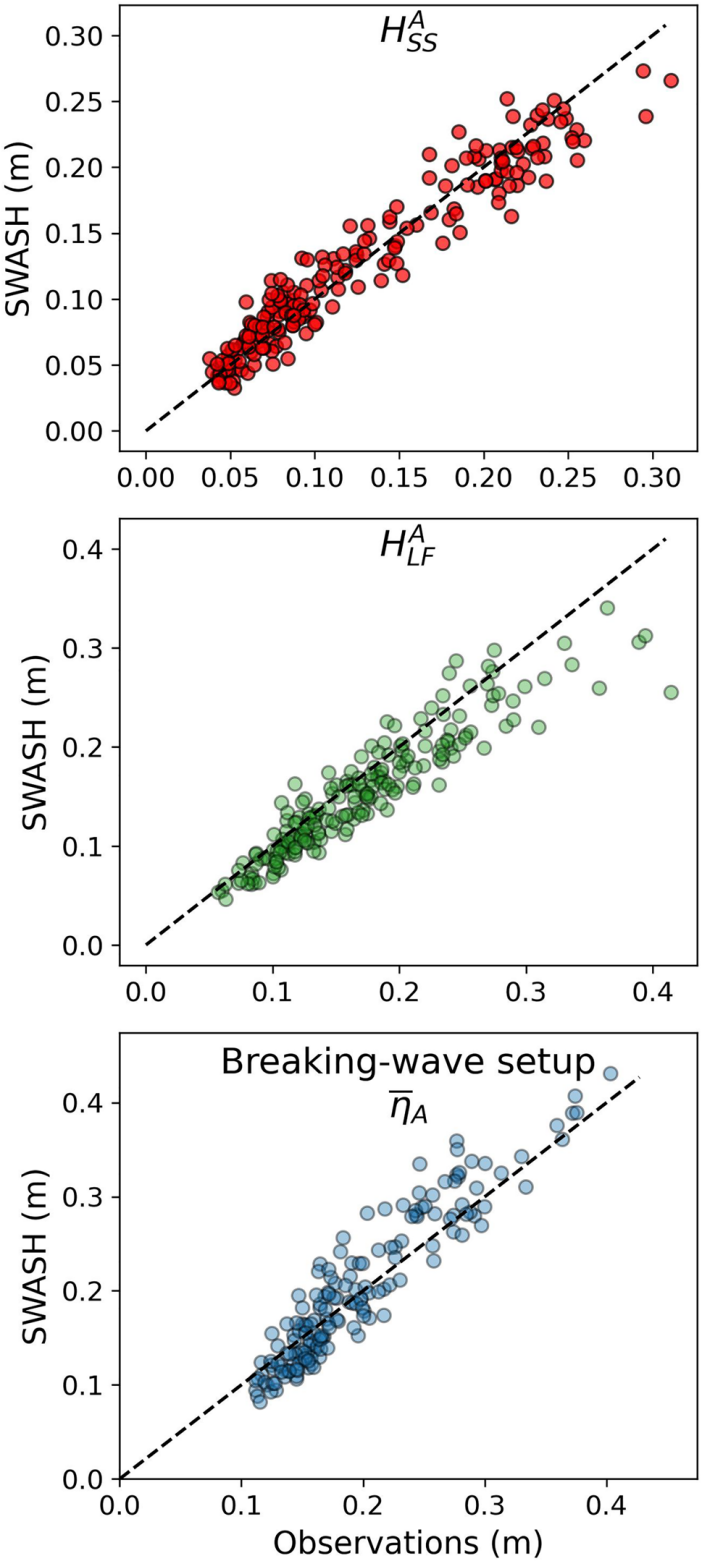
Model validation at the nearshore (sensor A) location of sea-swell significant wave height, low-frequency significant wave height, and breaking-wave setup.

To explore how this water level dependence of ${H}_{SS}$ will evolve with increases in water depth over the reef, we use a one-dimensional, phase-resolving nonhydrostatic numerical model, SWASH. Model simulations exhibit high skill (Fig. 4) in matching observed nearshore ${H}_{SS}$ with relatively low root-mean-square errors (${R}^{2}=0.93$, $RMSE=0.02 m$). SWASH simulations also do reasonably well in matching the observed nearshore LF significant wave height (${H}_{LF}$, ${R}^{2}=0.81$, $RMSE=0.03 m$) and breaking-wave setup (${\overline{\eta }}_{A}$, ${R}^{2}=0.78$, $RMSE=0.03 m$). We hypothesize that model results deviate from observations due to 2-dimensional effects not captured in our 1-dimensional simulations.

**Figure 5:**
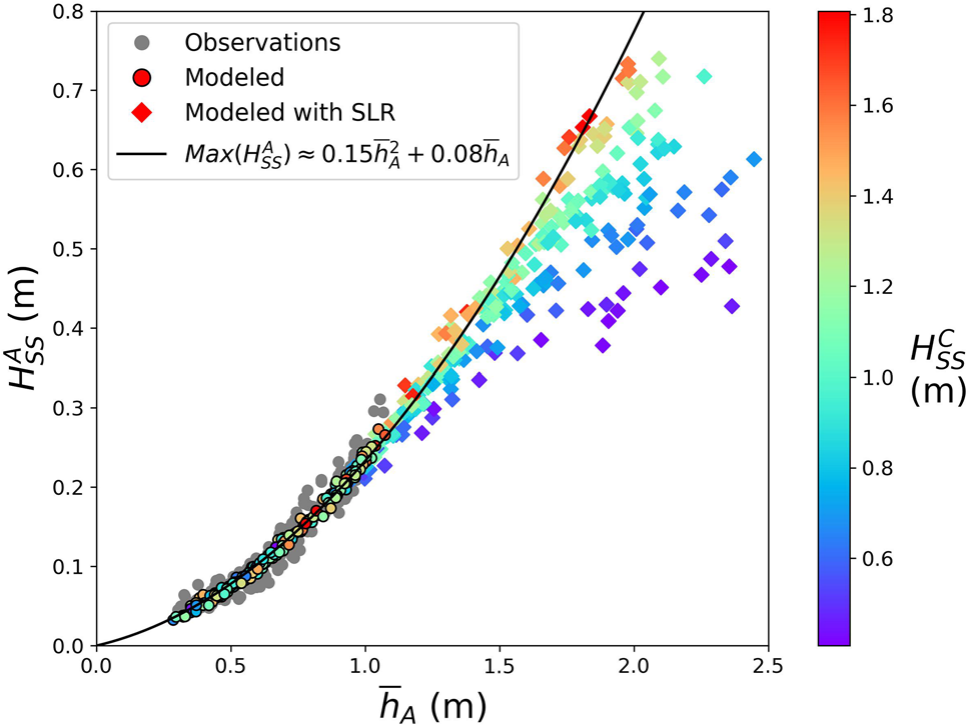
Modeled estimates of nearshore (sensor A) sea-swell significant wave height, ${H}_{SS}^{A}$, as a function of mean water depth nearshore, ${\overline{h} }_{A}$. 30-min average observed (gray dots) and modeled estimates of the nearshore sea-swell significant wave height as a function of total mean water depth nearshore, colored by the observed sea-swell significant wave height on the reef face (sensor C), ${H}_{SS}^{C}$. We estimate maximum nearshore significant wave height using a quadratic function of total reef flat water level. The regression is computed using all model results run with observed water levels and model results for which ${H}_{SS}^{C}>1.4$ m with added sea level.

Combining simulations for both observed and added sea levels, the model results suggest that the maximum nearshore ${H}_{SS}$ can be estimated using a quadratic function of nearshore mean water depth, ${\overline{h} }_{A}$, (Fig. 5) as

6 $${Max(H}_{SS}^{A})=0.15{\overline{h} }_{A}^{2}+0.08{\overline{h} }_{A} {\text{ }}({\text{m}}).$$

Above ~1.2 m mean water depth the scatter in shoreline SS wave heights increases, indicating greater dependence on offshore wave height, with only the highest waves reaching depth-limited breaking before the nearshore (sensor A) location. The coefficients in (6) are likely unique to our study site and sensitive to both reef bathymetry and friction. However, we hypothesize that the relationship is generalizable to similar reef-fringed coastlines.

**Figure 6:**
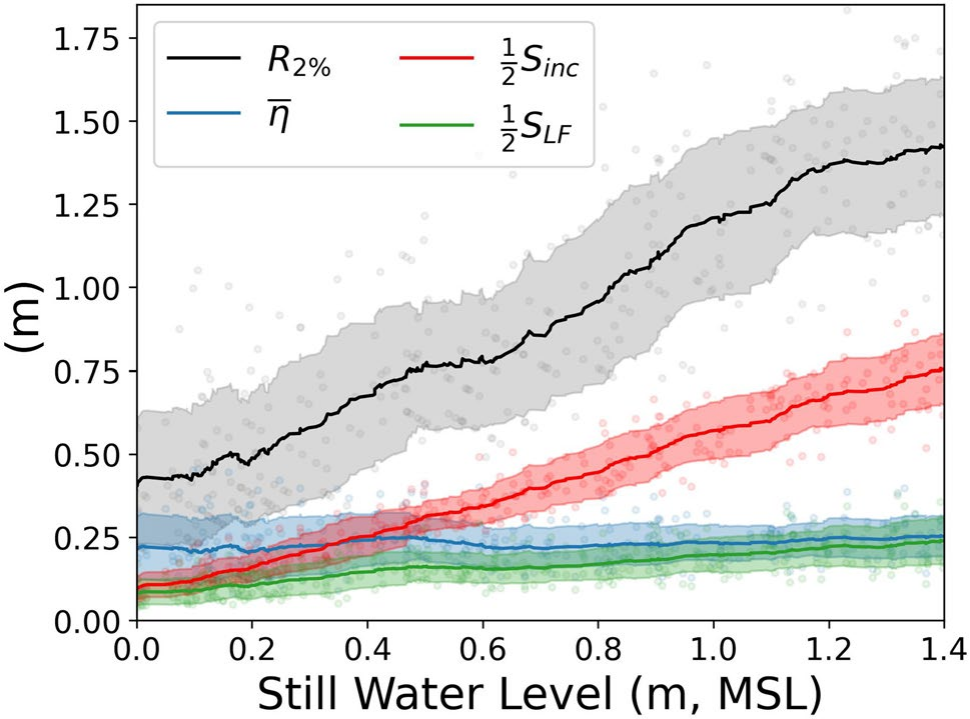
Modeled estimates of the 2% exceedance runup (${R}_{2\%}$) and individual contributions from breaking-wave setup ($\overline{\eta }$), significant swash heights in the sea-swell (${S}_{inc}$) and low-frequency (${S}_{LF}$) bands, as a function of still water level. Individual model runs are shown as dots, the running means of each quantity are shown using solid lines, and the shading indicates a running + /−1 standard deviation. Above 1.4 m MSL the coastal road is often inundated and runup statistics are confounded by the curvature of the roadside.

How would the increase in shoreline SS wave energy with rising sea levels impact coastal flooding? As an indicator of flood risk, we consider the wave-driven component of total water level (TWL), defined as the still water level plus the 2% exceedance runup elevation, ${R}_{2\%}$, computed directly from the model output time series. At Faga‘itua, the model indicates that greater sea levels will increase ${R}_{2\%}$ (Fig. 6). The rise in ${R}_{2\%}$ is dominated by the incident (sea-swell) swash (${S}_{inc}$) component. Breaking-wave setup at the shoreline ($\overline{\eta }$) remains relatively constant. The low frequency swash (${S}_{LF}$) component increases with rising sea level at a smaller rate than ${S}_{inc}$, suggesting that the LF energy is primarily set by nonlinear interactions of the shoaling swell. Uncertainty in modeled ${R}_{2\%}$ is not estimated, but Liu et al. (2021)^38^ found that laboratory measurements of ${R}_{2\%}$ on a fringing reef bathymetry matched SWASH estimates to within one standard deviation of model ensemble results.

**Figure 7:**
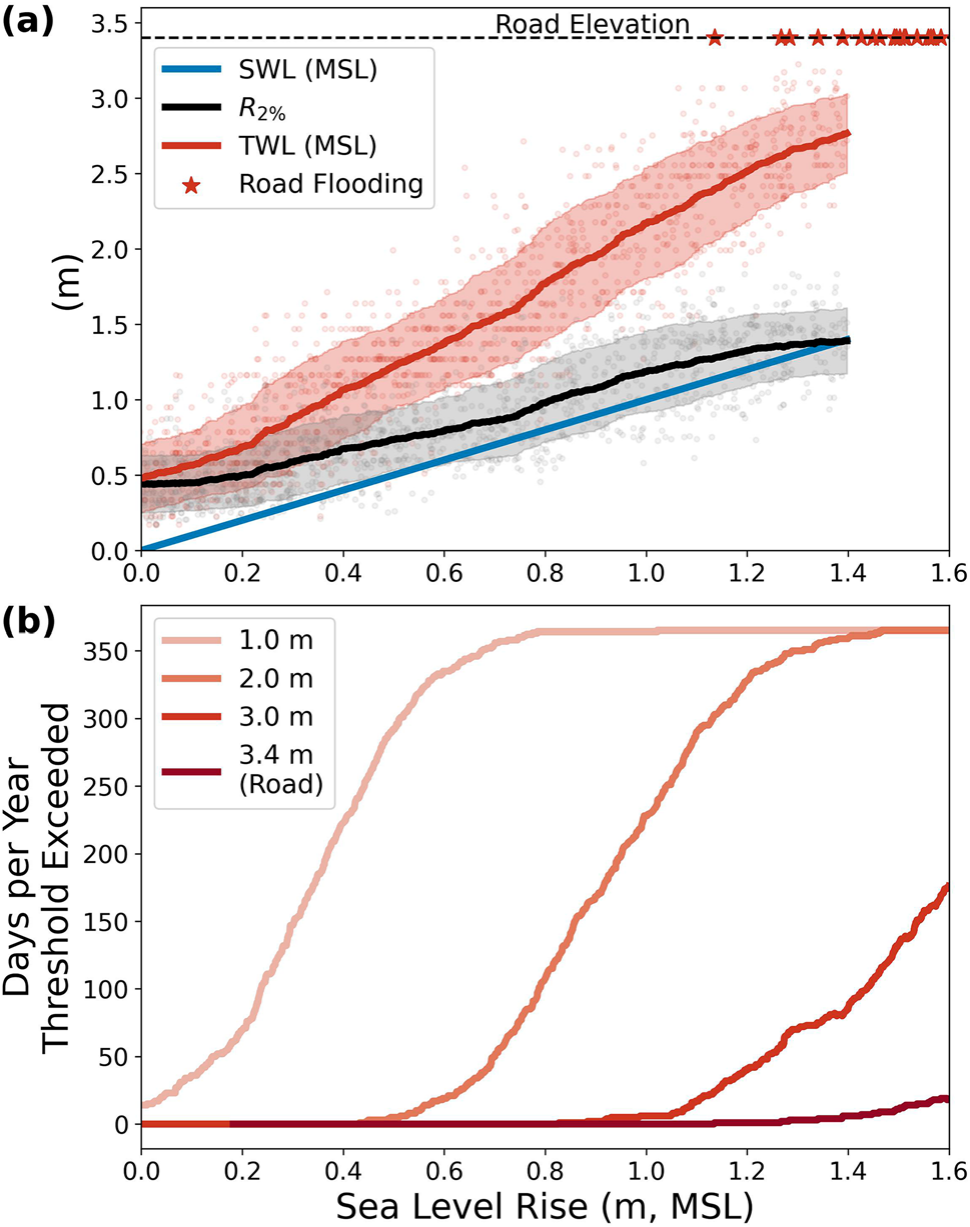
Modeled estimates of total water level (TWL), 2% exceedance runup (${R}_{2\%}$), and days per year of vertical threshold exceedance as a function of sea level rise. (**a**) Model runs (associated with 4 different values of sea level rise) are shown as dots, the running means of each quantity are shown using solid lines, and the shading indicates a running + /−1 standard deviation. Above 1.4 m MSL the coastal road is often inundated and runup statistics are confounded by the curvature of the roadside. (**b**) Days per year the TWL exceeds vertical thresholds at the shoreline. For each value of sea level rise, the percentage of model runs clustered around this value for which TWL exceeds the threshold is converted to days in a year.

The effects of SLR on coastal flood risk at Faga‘itua are twofold. Greater still water levels result in commensurate increases in wave-driven extreme runup (${R}_{2\%}$), leading to elevated total water levels that flood the coastal road at our study site consistently once SLR reaches 1.4 m (Fig. 7a), corresponding to relative SLR projections for 2100 under the intermediate emissions scenario^9^. We also estimate the number of days per year various vertical thresholds at the coastline are exceeded based on TWL (Fig. 7b).

**Figure 8:**
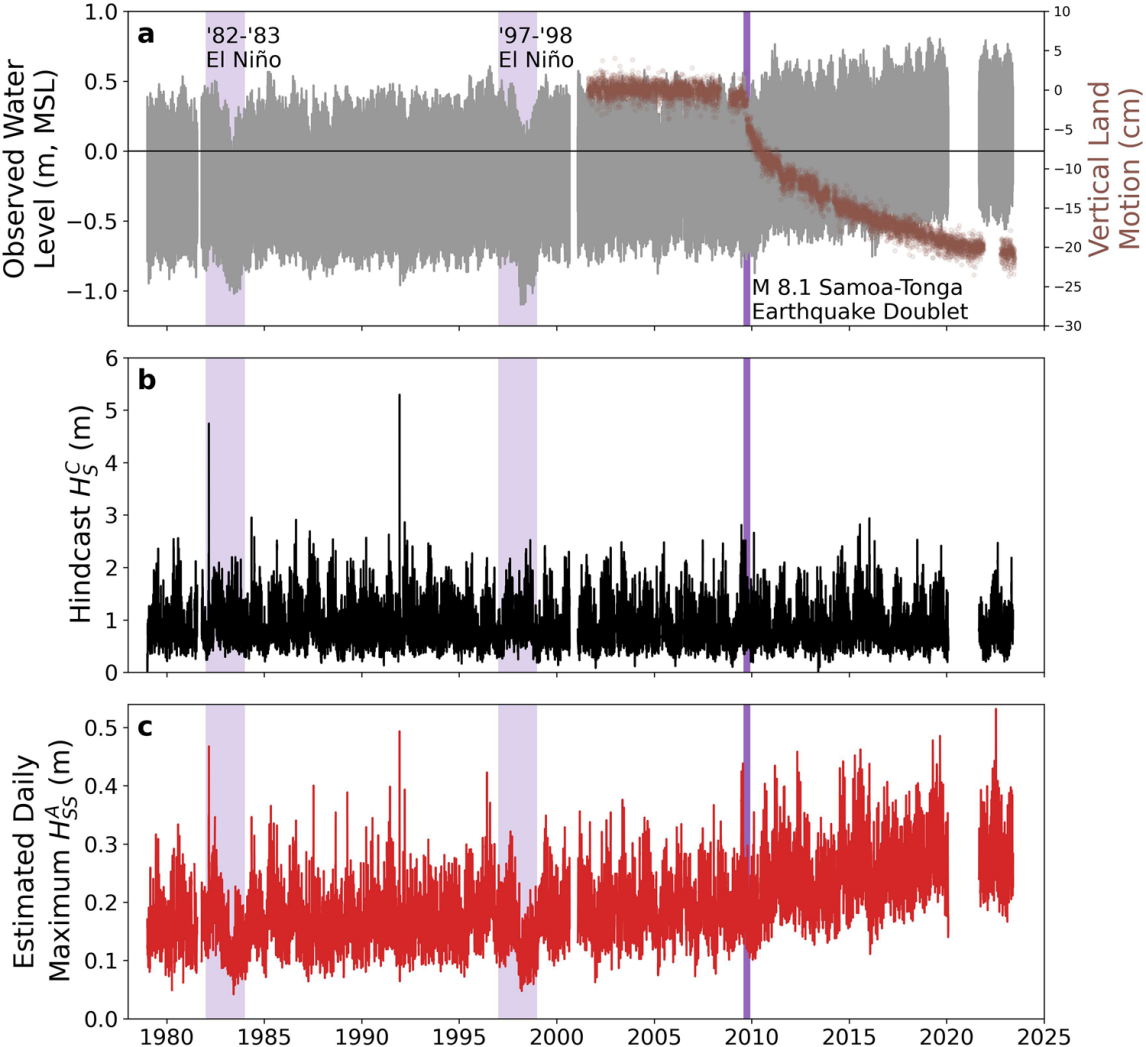
Historical sea level variability, hindcast reef face (sensor C) significant wave height, and daily maximum nearshore (sensor A) sea-swell significant wave height from 1979 through May 2023. (**a**) Observed sea level relative to mean sea level (MSL, tidal datum analysis period 01-Oct-2011 to 30-Sep-2019) at the Pago Pago tide gauge, courtesy of NOAA. We have highlighted the observed sea level depressions from the strong 1982–1983 and 1997–1998 El Niño events and indicated the magnitude 8.1 Samoa-Tonga Earthquake doublet on September 29, 2009. To highlight the rapid land subsidence following the 2009 earthquakes, vertical land motion from 2001 to present is shown on the right-hand vertical axis, relative to the pre-2009 mean; data courtesy of the Nevada Geodetic Laboratory^43^. (**b**) Bias-corrected hindcast reef face (sensor C) significant wave height using hindcasts from Smith et al. (2020)^36^ and a linear spectral refraction model. (**c**) Estimated daily maximum nearshore (sensor A) Faga‘itua sea-swell significant wave height based on the nearshore water depth.

We next estimate historical nearshore SS significant wave heights and how they have evolved with changes in relative sea level at Tutuila from 1979 through May 2023 (Fig. 8). Observed historical sea levels have, thus far, kept water levels at our observed Faga‘itua nearshore location below the 1.2 m threshold above which the model predicts nearshore SS wave height becomes increasingly dependent on offshore wave height. The mean water level is a combined estimate of sea level as measured by the NOAA Pago Pago tide gauge and the estimated breaking-wave setup. Our estimates of daily maximum nearshore SS significant wave height track closely with changes in relative sea level and show a marked increase as global sea levels have increased over several decades. Since 1979, the average of 0.32 m relative SLR has nearly doubled significant wave heights at the shoreline of Faga‘itua. El Niño events result in noticeably lower significant wave heights at the shoreline as regional water levels drop by 0.2–0.3 m^39^. A marked increase in coastal wave energy has occurred since the magnitude 8.1 Samoa-Tonga earthquake doublet.

## Discussion

Wave and water level observations at a fringing reef in Faga‘itua Bay on Tutuila island in American Samoa reveal that under current sea level, SS significant wave height at the shoreline is controlled by water depth over the reef, and the extreme wave-driven water levels are dominated by breaking-wave setup and LF motions. The greater the water level over the fringing reef, the greater the observed nearshore SS significant wave heights, consistent with depth-limited breaking. Deep water offshore wave heights determine the amount of breaking-wave setup, which increases the nearshore water level and therefore increases the nearshore SS wave height.

To assess how changes in relative sea level affect shoreline SS wave heights, we use SWASH, a phase-resolving nonhydrostatic numerical model applied at a cross-reef transect in Faga‘itua Bay. Waves are transformed over the fringing reef with past, present, and future water levels. We find that the model predicts that the maximum nearshore SS significant wave height for a given water depth increases quadratically with water depth. As water levels exceed ~1.2 m mean water depth, the dependence on offshore wave height increases as smaller waves are able to propagate across the reef without breaking.

Over the past 50 years, rising sea levels have contributed significantly to amplified shoreline wave energy at Faga‘itua Bay. From 1979 through September 2009, we estimate that the daily maximum nearshore SS significant wave height increased at a rate of 0.01 m/decade for an SLR of 0.04 m/decade; from October 2009 through May 2023, that rate jumped to 0.06 m/decade for an SLR of 0.16 m/decade. The effects of land subsidence associated with the earthquakes, most recently measured at 6 to 9 mm/yr^8^, are expected to continue for several decades^6^.

**Figure 9:**
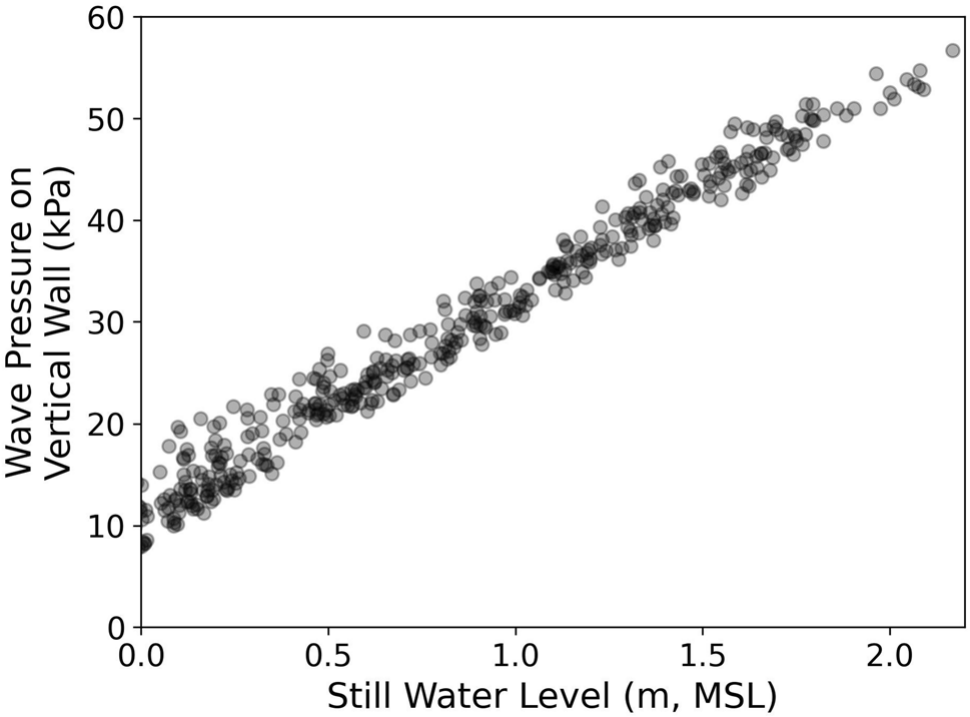
Modeled calculations of the maximum wave pressure on a hypothetical vertical breakwater constructed nearshore (sensor A), using the formulation from Goda (1974)^40^, as a function of still water level.

Modeling results suggest that the destructive potential of swells incident on fringing reef shorelines of American Samoa will increase as relative sea level increases. The extreme water level becomes dominated by SS wave heights as some SS waves cease to break at the reef crest. In addition to the flood risk associated with relative sea level increases projected for American Samoa, our results raise concerns about the flood risk associated with the increase in the 2% exceedance shoreline runup, and a growing dominance of SS energy. Given the proximity of built infrastructure to the shoreline at American Samoa, the combined impacts of increased waves and water levels pose a considerable challenge for coastal defense. For example, we use the SWASH results to illustrate how rising waves and water levels will impact the maximum wave pressure on a vertical breakwater (Fig. 9). We use Goda’s (1974)^40^ formulation for calculating the maximum wave pressure on a hypothetical vertical breakwater built nearshore (at sensor A’s location) as a function of sea level, assuming normal incidence. The maximum wave pressure, ${p}_{1}$ is a function of the significant wave height at A, ${H}_{S}^{A}$, the water depth, $h$, and the design wave wavelength, $L$ through the following adapted formulae:

7 $${p}_{1}=1.8\rho g{H}_{S}^{A}({\alpha }_{1}+\frac{2h}{1.8{H}_{S}^{A}})$$

8 $${\alpha }_{1}=0.6+\frac{1}{2}{\left(\frac{4h/L}{{\text{sinh}}(4\pi h/L)}\right)}^{2}$$

Where $\rho$ is the density of sea water, and $g$ is the gravitational acceleration. Our model suggests that as the nearshore water depth increases, the design wave height increases and therefore the maximum wave pressure on the vertical wall increases. Despite the increase in scatter of the nearshore significant wave height with increasing water depth, (7) predicts a near-linear increase in the maximum wave pressure.

Detailed analysis of future flooding is forthcoming and will incorporate the full suite of relative sea level projections developed for the American Samoa Sea Level Rise Viewer by Baizeau et al. (2023)^9^, estimates of future wave climatology, measured reef growth and accretion rates, and two-dimensional numerical modeling. This study assumes a fixed reef bathymetry, which we hope will serve as a possible worst-case scenario when it comes to reef response to SLR. Reef accretion and growth relative to rising sea level will limit increases in shoreline wave energy. Historical reef accretion rates inferred from cores taken at other reefs fringing Tutuila suggest that accretion rates have varied and kept pace with periods of rapid SLR during the Holocene. However, the species primarily responsible for this reef accretion appear to have died off in the past century, in part due to coastal construction in the 1940s^41^, motivating current reef growth and accretion study. Modeling 2-dimensional effects of alongshore reef variation will increase confidence in model results and spatial coverage of the projections, but we believe that the general results presented here will remain relevant. This study does not attempt to predict the timing of elevated water levels relative to the reef top but lays the groundwork for projections of nearshore SS wave energy once those estimates are constrained.

American Samoa’s relative sea level continues to rise, increasing the risk to coastal infrastructure from damaging wave events. The majority of American Samoa’s housing and infrastructure will face significant threats from the relative SLR and associated increases in nearshore SS wave energy. Coastal erosion rates have increased dramatically due to rapid relative SLR^42^, causing a cascade of deleterious impacts: loss of most sandy beaches around the island, loss of coastal land, damage to main roads, and the failures of seawalls that have hardened much of the coastline of Tutuila. Current passive flood modeling estimates based on relative sea level projections alone predict that much of American Samoa’s most critical infrastructure, including the only international airport and much of the main coastal road, will be threatened by 2100 under the intermediate emissions scenario resulting in 1.4 m relative SLR. Our model results suggest that the impacts under this amount of relative SLR will be greater because these estimates have not taken into account the increase in potentially destructive SS wave energy at the shoreline.

## Data Availability

The datasets generated and/or analyzed during the current study will be made publicly available through the UC San Diego Library and are currently available from the corresponding author on reasonable request. The preliminary DEM provided by NOAA is derived from aerial topographic/bathymetric (topobathy) light detection and ranging (LiDAR) measurements taken in 2022, and at the time of writing has not yet been through the full NOAA quality assurance process and approval. We assume liability of any further application of the data and errors present and are unable to share this data publicly at this time.
